# Profile of Co-Infection Prevalence and Antibiotics Use among COVID-19 Patients

**DOI:** 10.3390/pathogens11111250

**Published:** 2022-10-28

**Authors:** Rita Greco, Vittorio Panetta, Maria Teresa Della Rocca, Adriana Durante, Giovanni Di Caprio, Paolo Maggi

**Affiliations:** 1 UOSD Microbiology—AORN Sant’Anna and San Sebastiano, 81100 Caserta, Italy; 2 Infectious and Tropical Diseases Clinic, AORN Sant’Anna and San Sebastiano, 81100 Caserta, Italy; 3 Department of Infectious Disease, University of Campania Luigi Vanvitelli, 81100 Caserta, Italy

**Keywords:** COVID-19, bacterial co-infection, medical comorbidity data, antibiotic treatment

## Abstract

Bacterial co-infection in COVID-19 patients significantly contributes to the worsening of the prognosis based on morbidity and mortality. Information on the co-infection profile in such patients could help to optimize treatment. The purpose of this study was to describe bacterial co-infections associated with microbiological, clinical, and laboratory data to reduce or avoid a secondary infection. A retrospective cohort study was conducted at Sant’Anna and San Sebastiano Hospital from January 2020 to December 2021. Bacterial co-infection was detected in 14.3% of the COVID-19-positive patients. The laboratory findings on admission showed significant alterations in the median D-dimer, C-reactive protein, interleukin-6, and lactate dehydrogenase values compared to normal values. All inflammatory markers were significantly elevated. The most common pathogens isolated from blood cultures were *E. faecalis* and *S. aureus*. Instead, the high prevalence of respiratory tract infections in the COVID-19 patients was caused by *P. aeruginosa* (41%). In our study, 220 (82.4%) of the COVID-19 patients received antimicrobial treatment. Aminoglycosides and β-lactams/β-lactamase inhibitors showed the highest resistance rates. Our results showed that older age, underlying conditions, and abnormal laboratory parameters can be risk factors for co-infection in COVID-19 patients. The antibiotic susceptibility profile of bacterial pathogen infection provides evidence on the importance, for the clinicians, to rationalize and individualize antibiotic usage.

## 1. Introduction

The spread of coronavirus disease 2019 (COVID-19) in Italy prompted drastic measures for transmission containment; however, the infection continued to spread in our country, for which epidemiological data are continuously updated [1]. In several microbiological studies, vital respiratory infection has been shown to predispose patients to bacterial infection, often with a worse prognosis and mortality [2]. A recent report showed that approximately 50% of patients who died with COVID-19 had bacterial co-infections, which also increase the risk of mortality [3]. Several studies have evaluated bacterial and fungal co-infections in respiratory and blood specimens of hospitalized COVID-19 adults [4,5]. The most frequently isolated species in COVID-19 patients are *Mycoplasma pneumoniae*, *Staphylococcus aureus*, *Legionella pneumophila*, *Streptococcus pneumoniae*, *Haemophilus influenzae*, *Klebsiella* spp., *Mycobacterium tuberculosis*, and *Aspergillus* spp. [6]. Access in intensive care units is related to risk of infection, such as ventilator-associated pneumoniae (VAP), catheter-related urinary tract infection (UTI), sepsis, and septic shock [7,8]. The impact of bacterial co-infection on COVID-19 patients has been greatly investigated. Therefore, there is a need to study the effects of bacterial infections on COVID-19 patients to elucidate and determine their outcomes. Co-infected patients’ symptoms usually include fever, cough, dyspnea, diarrhea, and vomiting [9]. The clinical manifestations of the disease are mild, while in some subjects, it progresses to pneumonia, acute respiratory distress syndrome (ARDS), and multi-organ failure [10]. The significantly altered laboratory parameters were lymphopenia [10] and elevated D-dimer. C-reactive protein and inflammation parameters are predictive of coagulation-associated complications during hospitalization [11]. The symptoms of virus infection alone or bacterial co-infection are not always clear, so laboratory inflammatory markers associated with bacterial infection, such as high C-reactive protein, are significant in COVID-19 patients with bacterial co-infections [12]. In addition, these patients are more likely to demonstrate abnormalities upon chest RX (round glass opacities, consolidation, reticular pattern, or crazy paving pattern) [13] and, in some cases, admission to the intensive care unit [14]. Various therapies are used in both the community and hospital, including corticosteroids, low-molecular-weight heparins, and antibiotics [15,16]. There is no evidence on the use of antibiotic treatment in COVID-19 patients; however, antibiotics are being used in a lot of centers despite the low bacterial co-infection rates [16]. Several studies have reported antibiotically empirical treatment [17]. A combination of β-lactams and macrolides or fluoroquinolones is the main form used in hospitalized patients [18]. For this reason, throughout 2020, the phenomenon of antibiotic resistance has increased; as in most hospital settings, there has been a disproportionate use of broad-spectrum antibiotics that have not brought any benefits in terms of evolution and mortality of patients with severe COVID-19. The overuse of antibiotics and treatment can impact antimicrobial resistance. This study aimed to determine bacterial co-infection, associated with analysis of the determinant of co-infection through patient characteristics and microbiological and clinical laboratory data. In this way, we can optimize antimicrobial therapy and target antimicrobial stewardship interventions.

## 2. Materials and Methods

### 2.1. Study Design and Data Collection

A retrospective cohort study was conducted on COVID-19-positive patients admitted to AORN Sant’Anna and San Sebastiano of Caserta from January 2020 to December 2021. SARS-CoV-2 detection was performed using nasopharyngeal throat swabs via real-time PCR (RT-PCR) and an Xpert Xpress SARS-CoV-2 Kit (Cepheid, Sunnyvale, CA, USA). Negative COVID-19 pharyngeal swab test results were excluded. Demographic information and clinical characteristics (including medical history, underlying comorbidities, and symptoms and signs), laboratory findings, and chest RX scan results were obtained. Bacterial infection was also defined as those patients with a positive bacterial infection result from blood, respiratory, or urine culture tests with persistent clinically compatible symptoms during specimen collection. Microbiological data from all of the districts were collected in all patients at admission and during hospitalization, performed on usual media for blood culture, sputum, tracheal aspirate, plugged telescoping catheter, or bronchoalveolar lavage and urine, following the standard culture procedures. A pneumococcal and legionella urinary antigen test was used (BinaxNOW™ Legionella Urinary Antigen Card, Alere, Jouy-en-Josas, France; Strep Pneumo Meridian Bioscience, Cincinnati, Ohio,USA), and antimicrobial identification (MALDI-TOF MS, Biomèrieux, Marcy l’Étoile, France) and susceptibility (VITEKMS, Biomèrieux, Marcy l’Étoile, France) were also tested. The testing and identification of pathogens were carried out according to the guidelines of the European Committee on Antimicrobial Susceptibility Testing (EUCAST), along with breakpoint interpretation of the tested antibiotics.

### 2.2. Detection of Respiratory Pathogens

All COVID-19-positive patients were tested at admission for viral and bacterial co-infection with BioFire Respiratory Panel 2.1 Plus (BioFire, Salt Lake City, UT, USA). This test included respiratory pathogens: adenovirus; coronaviruses 229E, HKU1, NL63, OC43, Middle East respiratory syndrome coronavirus (MERS-CoV), and severe acute respiratory coronavirus 2 (SARS-CoV-2); human metapneumovirus; human rhinovirus/enterovirus; influenza A; influenza B; respiratory syncytial virus (RSV); and parainfluenza viruses 1, 2, 3, and 4. Bacterial detection revealed the eventual presence of *Bordetella parapertussis, Bordetella pertussis*, *Chlamydia pneumonia*e, and *Mycoplasma pneumoniae*.

### 2.3. Statical Analysis

Statistical analysis was conducted with IBM SPSS software (version 22.0; IBM SPSS Inc., New York, NY, USA). Descriptive statistics of the categorical variables are expressed as counts and percentages. The continuous variables are expressed as medians and interquartile ranges (IQRs). We used the Mann–Whitney *U*-test, chi-square test, and Fischer’s exact test to evaluate and compare differences between patients who had other infections and those who did not. A *p*-value of ≤0.05 was considered statistically significant.

## 3. Results

### 3.1. Baseline Demographic Characteristics of COVID-19 Patients

A total of 300 confirmed COVID-19 patients were admitted to the Infectious Disease Unit, Intensive Care Unit, and Pulmonary Department from January 2020 to December 2021. Males accounted for 69.2% of the admissions, and the average age of the patients was 63 years. Among the patients’ specific characteristics, higher comorbidity rates were observed for hypertension (45.7%), ischemic heart disease (25.7%), and diabetes mellitus (24.3%). The most common symptoms were fever (82.7%), dyspnea (83.1%), and cough (62.9%). Other symptoms included diarrhea (14.2%) and chest pain (2.9%). Furthermore, the length of hospitalization ranged from 5 to 15 days, with 12 days being the average (Table 1). From our data, 151 (56.5%) of the patients died from COVID-19, while those with bacterial co-infection accounted for 43 (14.3%) and those receiving antibiotic treatment accounted for 73.3% (220 of 300 patients).

### 3.2. Rate and Profile of Bacterial and Fungal Co-Infection in COVID-19 Patients

Table 1 reports on the 43 COVID-19 patients with bacterial co-infection compared to the non-co-infected COVID-19 patients. Based on the demographic and clinical characteristics, the co-infected patients had prolonged hospitalization stays (6–17 days) but were not significantly different. Higher comorbidity rates of hypertension (48.8%), diabetes mellitus (27.9%), obesity (13.9%), and smoking history (13.9%) were observed in the co-infected patients than in the total COVID-19 population. On the contrary, significantly lower rates for coronary insufficiency (11.6%), neoplasia (4.6%), and chronic kidney disease (6.9%) were observed in the co-infected patients compared to those of the non-co-infected COVID-19 patients. Of the total number of co-infected patients, during the hospitalization period, 86.0% had a fever and cough (67.4%), and higher rates of diarrhea (14.2%), vomiting (5.6%), and dyspnea (83.1%) were observed in the whole study population. Pharyngitis, anosmia, and/or dysgeusia were less common in the co-infected population.

In Table 2, we also report the standard laboratory and chest RX findings of the COVID-19 patients without bacterial co-infection on admission versus bacterial co-infected patients during hospitalization. On admission, hematological examination did not show abnormal white blood cells (median range = 7.4) or hemoglobin. The liver and renal function tests were normal. The coagulation factor range was still within the normal range. However, D-dimer, C-reactive protein, and lactate dehydrogenase (LDH) were altered compared to normal values, with a median range of 580, 9.09, and 790, respectively. Regarding bacterial co-infected patients, there were similarities in laboratory discoveries between white blood cells and hemoglobin and between liver and renal markers. Meanwhile, all of the inflammatory markers such as reactive protein, lactate dehydrogenase, and neutrophils were significantly elevated in patients with bacterial co-infection compared to patients without co-infection. Pulmonary involvement was checked by chest RX scans in patients without co-infection on admission and in patients with bacterial co-infection. In the first group, 41.4% of patients had bilateral interstitial pneumonia and 18.3% bilateral alveolar pneumonia. In the bacterial co-infected patients group, the pulmonary consolidation opacity rate was significantly higher for interstitial bilateral (52.2%) and alveolar bilateral (23.2%) pneumonia.

### 3.3. Profile of Viral and Bacterial Co-Infection in COVID-19 Patients

**Figure 1:**
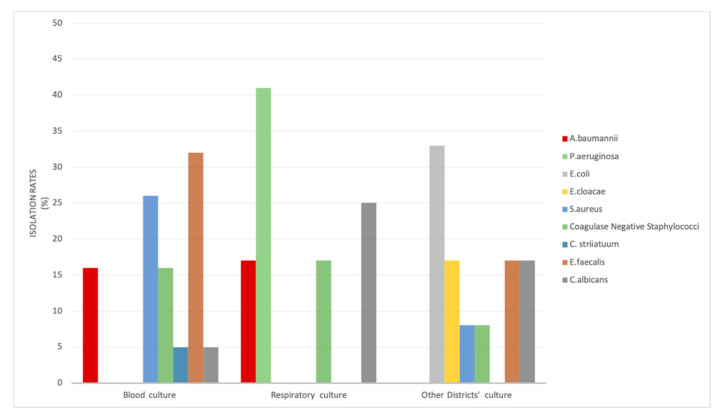
District frequency of the bacteria and yeast isolated from COVID-19 patients.

We described viral and bacterial co-infection in patients with COVID-19 pneumonia. SARS-CoV-2 infection was confirmed by multiplex real-time PCR in our study population. Nasopharyngeal swabs were analyzed to detect various respiratory pathogens. The viral targets were: influenza A; influenza B; parainfluenza types 1, 2, 3, and 4; human metapneumovirus; Middle East respiratory syndrome coronavirus (MERS); respiratory syncytial virus (RSV); human rhinovirus/enterovirus; adenovirus; and coronaviruses SARS-CoV-2, 229E, HKU1, NL63, and OC43. Bacteria such as *Bordetella pertussis, Bordetella parapertussis*, *Chlamydia pneumoniae,* and *Mycoplasma pneumoniae* were also sought. Among all of the COVID-19 patients, three were positive for SARS-CoV-2 and human rhinovirus/enterovirus, and one patient was positive for SARS-CoV-2 and parainfluenza virus 2. None of the patients presented co-infection with influenza A; this is related to similar epidemiological studies on COVID-19 and influenza [19]. The district frequency of bacterial infection in hospitalized COVID-19 patients is shown in Figure 1. Bacterial and fungal co-infection pathogens specifically relate to respiratory tract and bloodstream sources or other districts such as urine and medical devices. The overall percentage of co-infection pathogens of the study population was 14.3%, of which 44.1% were isolated from a bloodstream source. In this infection site, Gram-positive bacteria such as *E. faecalis, S. aureus,* and coagulase-negative staphylococci (Cons*)* were commonly isolated, while *A. baumannii* was the only Gram-negative bacterium (Figure 1), all of which was isolated from patients that recovered in the ICU. Instead, co-infections in respiratory tract specimens (28%) were high in prevalence caused by Gram-negative bacteria, particularly the *P. aeruginosa* strains (41%). The other districts showed an overall co-infection rate of 27.9% with *E. coli* (33%), *Enterococcus faecalis* (17%), and also *C. albicans* isolates (Figure 1). We observed the presence of fungaemia in approximately 14% of microbial co-infections.

Those study patients with bacterial co-infection showed differences in comorbidities and clinical and laboratory characteristics. We also assessed the distribution of bacterial co-infection in relation to gender and age. Similar pathogens in female patients compared with male have been reported [20]. Our data showed higher bacterial co-infection rates in males (74.4%) than females (25.6%). Regarding age, in the 48–58-year-old age group (*n* = 63), more *P. aeruginosa* was isolated. Then, in the 59–69-year-old age group (*n* = 110), *A. baumannii* infection was more prevalent. Finally, in the 70–80-year-old age group (*n* = 94), *E. faecalis* and *S. aureus* were mostly isolated.

**Figure 2:**
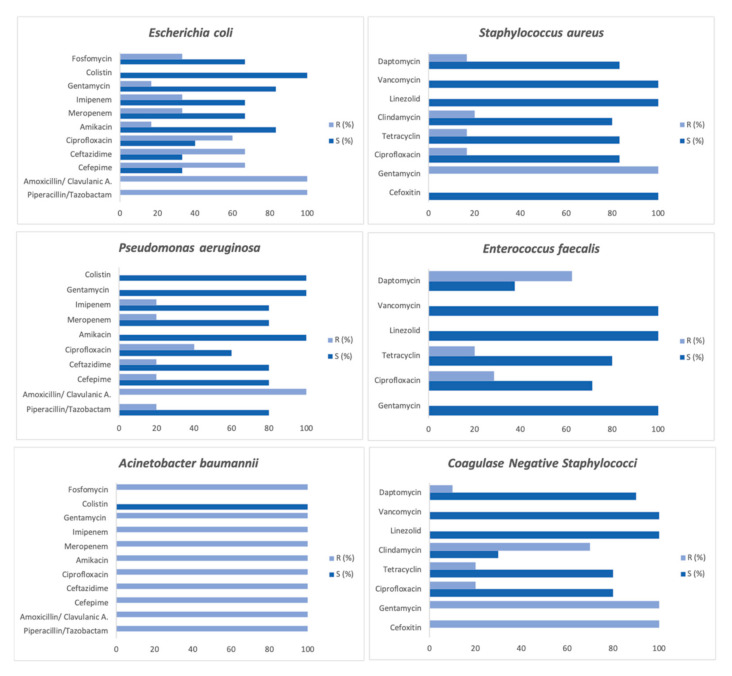
Antimicrobial susceptibility pattern of Gram-positive and Gram-negative strains isolated from COVID-19 patients.

Despite the low rate of bacterial and fungal co-infections in COVID-19 patients, high rates of antimicrobial prescription are reported [21]. In our study, 220 (73.3%) of SARS-CoV-2-positive patients received antimicrobial treatment. Based on the observations in this study, antibiotics were commonly prescribed during the first wave of the COVID-19 pandemic, following local empirical treatment guidelines. In order to report the impact of antibiotics use in COVID-19 patients on antimicrobial resistance, we reported the susceptibility pattern of antimicrobial agents of Gram-positive (48.8%) and Gram-negative (37.2%) isolates from COVID-19 patients. The results showed higher resistance rates in the *E. coli* and *A. baumannii* isolates. All of the Gram-negative isolates were resistant to amoxicillin/clavulanic acid (100%) and piperacillin/tazobactam (100%) and sensible to colistin (100%) (Figure 2). The *P. aeruginosa* isolates showed the highest susceptibility rates for cephalosporins (80%) and amikacin (100%). The *S. aureus* strains were all susceptible to cefoxitin (100%), while coagulase-negative staphylococci were all resistant. All of the Gram-positive isolates from the COVID-19 patients were susceptible to linezolid and vancomycin. We also reported the resistance rates of daptomycin (62.5%) in the *E. faecalis* strains. This susceptibility pattern reflects the antibiotics guidelines for administration to COVID-19 patients. In fact, a common combination of antibiotics with broad-spectrum coverage (either the amoxicillin/clavulanate or piperacillin/tazobactam combination) was used [22].

## 4. Discussion

The prevalence of bacterial co-infection in confirmed COVID-19 patients significantly contributes to the worsening of the prognosis based on morbidity and mortality [23,24]. In 267 COVID-19-positive patients admitted from January 2020 to December 2021 to AORN of Sant’Anna and San Sebastiano Caserta, a rate of bacterial infection of 14.3% was detected. This proportion is higher compared to that of Langford et al., who confirmed a bacterial infection prevalence of 8.0% [25]. The majority of the patients were male (69.2%), with an average age of 63 years old, despite several studies reporting a median age of approximately 50 years [26]. The highest comorbidities in this study population were due to hypertension (45.7%), ischemic heart disease (25.7%), and diabetes mellitus (24.3%), as previously observed by Wu et al. in 2020 [27]. The co-infected population compared to the total COVID-19-positive population showed significantly high comorbidity rates for diabetes mellitus, hypertension, obesity, and smoking history. On the contrary, significantly lower rates for coronary insufficiency, neoplasy, and chronic kidney disease were observed in the co-infected patients instead of the COVID-19-positive patients [28]. Pulmonary involvement was checked via chest RX scan primarily in the admitted patients and after in the patients with bacterial co-infection. The pulmonary consolidation opacity rate in bacterial co-infected patients was significantly higher for interstitial bilateral and alveolar bilateral [29]. Inflammation parameters are elevated in cases of bacterial co-infection [30]. The co-infected patients in this study showed alteration in several inflammatory markers, such as reactive protein, lactate dehydrogenase, procalcitonin, and neutrophil, which increased significantly. A high neutrophil count indicates an imbalance in the inflammatory response from increased neutrophil and decreased lymphocyte counts [31]. Meanwhile, laboratory parameters can predict COVID-19 prognosis and bacterial co-infection through C-reactive protein and procalcitonin [32]. Microbiological samples were collected from all patients using culture tests and rapid molecular screening to identify 21 different bacteria species. This study discovered that in blood cultures, the most common bacterial co-infection pathogens were *S. aureus* and *E. faecalis*, followed by *A. baumannii*. Bacterial co-infection of respiratory specimens was mostly caused by *A. baumannii* and *P. aeruginosa*. In the current study, we observed the presence of fungaemia in approximately 14% of microbial co-infections. Higher co-infection rates and pathogen levels are more commonly found in females compared to males. Regarding age, *P. aeruginosa* infection was more frequently found in the 48–58-year-old group than in the 59–69-year-old age group, which experienced *A. baumannii* co-infection more frequently. At admission, none of COVID-19 co-infected patients presented positive blood cultures or multidrug-resistant organism (MDRO) colonization. Bacterial epidemiology infection is closely linked to hospital-acquired infection pathogens and ICU admission [33], as in our reality. Despite low bacterial co-infection in COVID-19 patients, high resistance rates of antimicrobial agents were reported. This is related to the fact that a higher proportion of patients treated with antibiotics do not have suspected or confirmed bacterial infections [34]. Our study showed that antibiotics were empirically administered in 73.3% of COVID-19 co-infected patients. This is similar to Musuuza et al.’s study, in which it was reported that 98% of the administered empirical antibiotic, aminoglycoside, and quinolone antibiotic classes demonstrated the highest resistance rates [35]. The antibiotic susceptibility pattern shown in our study is in line with antibiotic usage in COVID-19 patients [36,37]. The *A. baumannii* and *E. coli* isolated strains showed resistance to the beta lactam class and quinolones rather than susceptibility to colistin. The highest resistance rates for gentamycin and susceptibility to vancomycin and linezolid were reported for Gram-positive bacteria. The limitation of this study is that it included only data collected from a single hospital in southern Italy, and it lacked a larger patient cohort. However, our hospital is the fulcrum in emergency management in provincial reality. Providing health services in ordinary hospitalization, first aid, day hospitals, and even outpatients allowed us to include in our analysis a very heterogeneous group of patients. Further studies, with a wider patient cohort, are needed to increase our knowledge about the prevalence and risks of COVID-19 bacterial co-infection, as well as the influence of empirical antibiotics usage on bacterial resistance.

## 5. Conclusions

With the pressure on the healthcare system during the COVID-19 pandemic, a general evidence base on bacterial co-infection with a focus on antimicrobial prescribing is required to support optimal treatment and prevent the consequences of antimicrobial misuse. This study showed that older age, longer length of hospital stays, diabetes mellitus, hypertension, obesity, smoking history, and higher neutrophil, C-reactive protein, and procalcitonin are value risk factors for co-infection in COVID-19 patients. The antibiotic susceptibility profile provides evidence on the importance, for the clinicians, to rationalize and individualize antibiotic use. Consequently, antimicrobial stewardship efforts are crucial to reduce unnecessary antibiotic exposure and alleviate the adverse impact on antimicrobial resistance during the pandemic era.

## Figures and Tables

**Table 1:** Demographic and clinical characteristics of COVID-19 patients with and without co-infection.

Variables	COVID-19 Patients without Co-Infection *n* = 267	COVID-19 Patients with Co-Infection *n* = 43	*p*-Value
**Gender % (n)**			
Male	69.2 (185)	74.4 (32)	0.043 *
Female	30.8 (82)	25.6 (11)	
Mean age, years	63.3 (48–80)	61.2 (50–77)	0.975
Length of hospitalization (median,IQR), days	12 (5–15)	14 (6–17)	0.522
**Any comorbidities % (*n*)**			
Hypertension	45.7 (122)	48.8 (21)	0.865
Ischemic Heart Disease	25.8 (69)	25.6 (11)	0.989
Diabetes Mellitus	24.3 (65)	27.9 (12)	0.517
Coronary Insufficiency	18.3 (49)	11.6 (5)	0.241
Smoking history	11.6 (31)	13.9 (6)	0.062 *
Obesity	10.1 (27)	13.9 (6)	0.035 *
Pulmonary diseases	8.6 (23)	11.6 (5)	0.412
Chronic kidney disease	7.1 (19)	6.9 (3)	0.908
Neoplasy	5.9 (16)	4.6 (2)	0.476
Cirrose	0 (0)	0 (0)	-
Human Immunodeficiency Virus	0 (0)	0 (0)	-
**Symptoms**			
Fever	82.7 (221)	86.0 (37)	0.123
Dyspnea	83.1 (222)	81.4 (35)	0.563
Cough	62.9 (168)	67.4 (29)	0.346
Diarrhea	14.2 (38)	11.6 (5)	0.322
Vomiting	5.6 (15)	4.6 (2)	0.645
Chest pain	2.9 (8)	2.3 (1)	0.675
Pharyngitis	2.6 (7)	2.3 (1)	0.908
Anosmia and/or Dysgeusia	1.5 (4)	2.3 (1)	0.238
Dizziness	0 (0)	-	-
Rhinorrhea	0 (0)	-	-
Asymptomatic	2.2 (6)	-	-
**Signs**			
Systolic arterial pressure, mm Hg	132.4 (120–140)	110 (110–120)	0.509
Diastolic arterial pressure, mm Hg	76 (70–80)	70 (70–80)	0.873
Heart rate, bpm	85.8 (27.2–92)	80 (30–90)	0.242
Respiratory rate, rpm	21.3 (17.5–24.5)	22.8 (21–23)	0.482

* *p*-value ≤ 0.05 was considered statistically significant.

**Table 2:** Laboratory and radiologic findings of COVID-19 patients.

Parameters	Primarily Admitted Patients without Co-Infection	Patients with Bacterial Co-Infection	*p*-Value
Laboratory Findings	Median (Interquartile Range)		
N-terminal prohormone of brain natriuretic peptide (Pro-BNP)	917.1 (199.9–2977.75)	921 (200–29766.87)	0.785
Lactate Dehydrogenase, UI/I (Normal range 125–250)	790 (576–903)	920 (624–989)	0.012 *
D-Dimer, % (Normal range 0–250)	580 (296–2590.5)	572 (287–2665.4)	0.976
Thrombocytes, %	186 (131–259)	183 (132–262)	0.991
Blood Glucose Level, mg/dL (Normal range 76–110)	124 (104–153)	130 (102–170)	0.891
Creatine phosphokinase, UI/I (Normal range 0–200)	112 (48.5–231.5)	110 (47.2–230.1)	0.997
Neutrophil, % (Normal range 40–68)	80.05 (71.43–87.3)	90.4 (80.21–92.8)	0.032 *
Myoglobin	77.1 (33.66–258.9)	81.8 (34.88–260.2)	0.795
Interleukin-6, g/mL (Normal range 0–70)	59.37 (17.82–80.31)	60.12 (17.34–79.21)	0.871
Aspartate aminotransferase (AST), UI/I (Normal range 0–41)	39 (28.5–62.5)	38.2 (27.1–62.7)	0.972
Alanine aminotransferase (ALT), UI/I (Normal range 0–40)	31 (19–53)	29 (19–52)	0.871
Activated partial thromboplastin time (aPTT), (Normal range 25–38)	31 (28–34)	34 (28–35)	0.890
Azotemia, Blood Urea Nitrogen (Normal range 5–25)	18 (12.5–26)	17 (12.5–2.8)	0.971
Hemoglobin, g/L (Normal range 14–18)	13.6 (11.6–14.6)	15 (10.4–16.5)	0.995
Lymphocyte, % (Normal range 20–45)	13.5 (7.6–18.8)	19.8 (8.9–21.0)	0.037 *
C-reactive Protein, mg/dL (Normal range 0–0.5)	9.09 (3.02–16.28)	10.9 (4.03–18.07)	0.091
White Blood Cell, ×10^9^/L (Normal range 4.2–10)	7.4 (5.17–11.26)	7.8 (5.23–11.78)	0.923
International Normalized Ratio (INR), (Normal range 0.8–1.2)	1.19 (1.09–1.3)	1.17 (1.00–1.2)	1.458
Procalcitonin, ng/mL (Normal range 0–0.05)	0.2 (0.083–0.78)	2.3 (0.2–3.5)	0.081
Troponin-I, ng/mL (Normal range 0.10–0.15)	0.11 (0.1–0.14)	0.14 (0.1–0.15)	0.872
Albumin, g/dL (Normal range 3.5–5.0)	3.1 (2.7–3.4)	3.7 (2.5–3.6)	0.975
Creatinine, mg/dL (Normal range 0.7–1.2)	0.9 (0.67–1.1)	1.0 (0.70–1.2)	0.998
Bilirubin Total, mg/dL (Normal range 0–1.1)	0.5 (0.4–0.6)	0.5 (0.4–0.6)	-
Bilirubin direct, mg/dL	0.2 (0.2–0.3)	0.2 (0.2–0.3)	-
Chest RX Image Finding *	**% (n)**		
Interstitial Bilateral	41.4 (111)	51.2 (22)	0.021 *
Alveolar Bilateral	18.3 (49)	23.2 (10)	0.035 *
Interstitial monolateral	12.7 (34)	13.9 (6)	0.342
Alveolar monolateral	10.1 (27)	13.9 (6)	0.432
Alveolar interstitial Bilateral lung	7.1 (19)	11.6 (5)	0.082
Normal	5.6 (15)	4.6 (2)	0.642
Alveolar Interstitial	2.6 (7)	4.6 (2)	0.062
Alveolar Interstitial Monolateral	1.5 (4)	2.3 (1)	0.231

* *p*-value ≤ 0.05 was considered statistically significant.

## References

[1] Gatto M., Bertuzzo E., Mari L., Miccoli S., Carraro L., Casagrandi R., Rinaldo A. (2020). Spread and dynamics of the COVID-19 epidemic in Italy: Effects of emergency containment measures. Proc. Natl. Acad. Sci. USA.

[2] Feldman C., Anderson R. (2021). The role of co-infections and secondary infections in patients with COVID-19. Pneumonia (Nathan Qld.).

[3] Arnold F.W., Fuqua J.L. (2020). Viral respiratory infections: A cause of community-acquired pneumonia or a predisposing factor?. Curr. Opin. Pulm. Med..

[4] Chong W.H., Saha B.K., Ramani A., Chopra A. (2021). State-of-the-art review of secondary pulmonary infections in patients with COVID-19 pneumonia. Infection.

[5] Fattorini L., Creti R., Palma C., Pantosti A. (2020). Unit of Antibiotic Resistance and Special Pathogens; Unit of Antibiotic Resistance and Special Pathogens of the Department of Infectious Diseases, Istituto Superiore di Sanità, Rome. Bacterial coinfections in COVID-19: An underestimated adversary. Ann. Ist. Super. Sanita.

[6] Lansbury L., Lim B., Baskaran V., Lim W.S. (2020). Co-Infections in People with COVID-19: A Systematic Review and Meta-Analysis. J. Infect..

[7] Chotpitayasunondh T., Fischer T.K., Heraud J.M., Hurt A.C., Monto A.S., Osterhaus A., Shu Y., Tam J.S. (2021). Influenza and COVID-19: What does co-existence mean?. Influenza Other Respir. Viruses.

[8] Rossato L., Negrão F.J., Simionatto S. (2020). Could the COVID-19 pandemic aggravate antimicrobial resistance?. Am. J. Infect. Control..

[9] Loomba R.S., Aggarwal G., Aggarwal S., Flores S., Villarreal E.G., Farias J.S., Lavie C.J. (2021). Disparities in case frequency and mortality of coronavirus disease 2019 (COVID-19) among various states in the United States. Ann. Med..

[10] Singhal T. (2020). A Review of Coronavirus Disease-2019 (COVID-19). Indian J. Pediatr..

[11] Gao Y.D., Ding M., Dong X., Zhang J.J., Kursat Azkur A., Azkur D., Gan H., Sun Y.L., Fu W., Li W. (2021). Risk factors for severe and critically ill COVID-19 patients: A review. Allergy.

[12] Skevaki C., Fragkou P.C., Cheng C., Xie M., Renz H. (2020). Laboratory characteristics of patients infected with the novel SARS-CoV-2 virus. J. Infect..

[13] Chen N., Zhou M., Dong X., Qu J., Gong F., Han Y., Qiu Y., Wang J., Liu Y., Wei Y. (2020). Epidemiological and clinical characteristics of 99 cases of 2019 novel coronavirus pneumonia in Wuhan, China: A descriptive study. Lancet.

[14] Ye Z., Zhang Y., Wang Y., Huang Z., Song B. (2020). Chest CT manifestations of new coronavirus disease 2019 (COVID-19): A pictorial review. Eur. Radiol..

[15] Stasi C., Fallani S., Voller F., Silvestri C. (2020). Treatment for COVID-19: An overview. Eur. J. Pharmacol..

[16] Beović B., Doušak M., Ferreira-Coimbra J., Nadrah K., Rubulotta F., Belliato M., Berger-Estilita J., Ayoade F., Rello J., Erdem H. (2020). Antibiotic use in patients with COVID-19: A ‘snapshot’ Infectious Diseases International Research Initiative (ID-IRI) survey. J. Antimicrob. Chemother..

[17] Rawson T.M., Moore L., Castro-Sanchez E., Charani E., Davies F., Satta G., Ellington M.J., Holmes A.H. (2020). COVID-19 and the potential long-term impact on antimicrobial resistance. J. Antimicrob. Chemother..

[18] Rawson T.M., Moore L., Zhu N., Ranganathan N., Skolimowska K., Gilchrist M., Satta G., Cooke G., Holmes A. (2020). Bacterial and Fungal Coinfection in Individuals with Coronavirus: A Rapid Review to Support COVID-19 Antimicrobial Prescribing. Clin. Infect. Dis..

[19] Ozaras R., Cirpin R., Duran A., Duman H., Arslan O., Bakcan Y., Kaya M., Mutlu H., Isayeva L., Kebanlı F. (2020). Influenza and COVID-19 coinfection: Report of six cases and review of the literature. J. Med. Virol..

[20] Chen X., Liao B., Cheng L., Peng X., Xu X., Li Y., Hu T., Li J., Zhou X., Ren B. (2020). The microbial coinfection in COVID-19. Appl. Microbiol. Biotechnol..

[21] Langford B.J., So M., Raybardhan S., Leung V., Soucy J.R., Westwood D., Daneman N., MacFadden D.R. (2021). Antibiotic prescribing in patients with COVID-19: Rapid review and meta-analysis. Clin. Microbiol. Infect..

[22] Vaughn V.M., Gandhi T.N., Petty L.A., Patel P.K., Prescott H.C., Malani A.N., Ratz D., McLaughlin E., Chopra V., Flanders S.A. (2021). Empiric Antibacterial Therapy and Community-onset Bacterial Coinfection in Patients Hospitalized With Coronavirus Disease 2019 (COVID-19): A Multi-hospital Cohort Study. Clin. Infect. Dis..

[23] Albitar O., Ballouze R., Ooi J.P., Sheikh Ghadzi S.M. (2020). Risk factors for mortality among COVID-19 patients. Diabetes Res. Clin. Pract..

[24] Arastehfar A., Carvalho A., Nguyen M.H., Hedayati M.T., Netea M.G., Perlin D.S., Hoenigl M. (2020). COVID-19-Associated Candidiasis (CAC): An Underestimated Complication in the Absence of Immunological Predispositions?. J. Fungi.

[25] Langford B.J., So M., Raybardhan S., Leung V., Westwood D., MacFadden D.R., Soucy J.R., Daneman N. (2020). Bacterial co-infection and secondary infection in patients with COVID-19: A living rapid review and meta-analysis. Clin. Microbiol. Infect..

[26] Vincent J.L., Rello J., Marshall J., Silva E., Anzueto A., Martin C.D., Moreno R., Lipman J., Gomersall C., Sakr Y. (2009). International study of the prevalence and outcomes of infection in intensive care units. JAMA.

[27] Wu Z., McGoogan J.M. (2020). Characteristics of and Important Lessons From the Coronavirus Disease 2019 (COVID-19) Outbreak in China: Summary of a Report of 72 314 Cases From the Chinese Center for Disease Control and Prevention. JAMA.

[28] Sanyaolu A., Okorie C., Marinkovic A., Patidar R., Younis K., Desai P., Hosein Z., Padda I., Mangat J., Altaf M. (2020). Comorbidity and its Impact on Patients with COVID-19. SN Compr. Clin. Med..

[29] Silva D.L., Lima C.M., Magalhães V., Baltazar L.M., Peres N., Caligiorne R.B., Moura A.S., Fereguetti T., Martins J.C., Rabelo L.F. (2021). Fungal and bacterial coinfections increase mortality of severely ill COVID-19 patients. J. Hosp. Infect..

[30] Ponti G., Maccaferri M., Ruini C., Tomasi A., Ozben T. (2020). Biomarkers associated with COVID-19 disease progression. Crit. Rev. Clin. Lab. Sci..

[31] Garcia-Vidal C., Sanjuan G., Moreno-García E., Puerta-Alcalde P., Garcia-Pouton N., Chumbita M., Fernandez-Pittol M., Pitart C., Inciarte A., Bodro M. (2021). Incidence of co-infections and superinfections in hospitalized patients with COVID-19: A retrospective cohort study. Clin. Microbiol. Infect..

[32] Heer R.S., Mandal A.K., Kho J., Szawarski P., Csabi P., Grenshaw D., Walker I.A., Missouris C.G. (2021). Elevated procalcitonin concentrations in severe Covid-19 may not reflect bacterial co-infection. Ann. Clin. Biochem..

[33] Nebreda-Mayoral T., Miguel-Gómez M.A., March-Rosselló G.A., Puente-Fuertes L., Cantón-Benito E., Martínez-García A.M., Muñoz-Martín A.B., Orduña-Domingo A. (2020). Bacterial/fungal infection in hospitalized patients with COVID-19 in a tertiary hospital in the Community of Castilla y León, Spain. Enferm. Infect. Microbiol. Clin..

[34] Segala F.V., Bavaro D.F., Di Gennaro F., Salvati F., Marotta C., Saracino A., Murri R., Fantoni M. (2021). Impact of SARS-CoV-2 Epidemic on Antimicrobial Resistance: A Literature Review. Viruses.

[35] Musuuza J.S., Watson L., Parmasad V., Putman-Buehler N., Christensen L., Safdar N. (2021). Prevalence and outcomes of co-infection and superinfection with SARS-CoV-2 and other pathogens: A systematic review and meta-analysis. PLoS ONE.

[36] Mohamad I.N., Wong C.K., Chew C.C., Leong E.L., Lee B.H., Moh C.K., Chenasammy K., Lim S.C., Ker H.B. (2022). The landscape of antibiotic usage among COVID-19 patients in the early phase of pandemic: A Malaysian national perspective. J. Pharm. Policy Pract..

[37] Jean S.S., Lee P.I., Hsueh P.R. (2020). Treatment options for COVID-19: The reality and challenges. J. Microbiol. Immunol. Infect..

